# Legal sourcing of ten cannabis products in the Canadian cannabis market, 2019–2021: a repeat cross-sectional study

**DOI:** 10.1186/s12954-023-00753-6

**Published:** 2023-02-17

**Authors:** Elle Wadsworth, Vicki Rynard, Pete Driezen, Tom P. Freeman, Marta Rychert, Chris Wilkins, Wayne Hall, Robert Gabrys, David Hammond

**Affiliations:** 1 School of Public Health Sciences, University of Waterloo, 200 University Ave W, Waterloo, ON N2L 3G1 Canada; 2 Canadian Centre on Substance Use and Addiction, 75 Albert St, Suite 500, Ottawa, ON K1P 5E7 Canada; 3 Department of Psychology, University of Waterloo, 200 University Ave W, Waterloo, ON N2L 3G1 Canada; 4 Addiction and Mental Health Group (AIM), Department of Psychology, University of Bath, Bath, BA2 7AY UK; 5 SHORE & Whāriki Research Centre, College of Health, Massey University, Palmerston North, New Zealand; 6 National Centre for Youth Substance Use Research, The University of Queensland, St Lucia, Australia; 7 Present Address: RAND Europe, Westbrook Centre, Milton Road, Cambridge, CB4 1YG UK

**Keywords:** Cannabis, Marijuana, Legalization, Sourcing, Legal markets

## Abstract

**Background:**

One of the objectives of cannabis legalization in Canada is to transition consumers from the illegal to the legal market. Little is known about how legal sourcing varies across different cannabis product types, provinces, and frequency of cannabis use.

**Methods:**

Data were analyzed from Canadian respondents in the International Cannabis Policy Study, a repeat cross-sectional survey conducted annually from 2019 to 2021. Respondents were 15,311 past 12-month cannabis consumers of legal age to purchase cannabis. Weighted logistic regression models estimated the association between legal sourcing (“all”/ “some”/ “none”) of ten cannabis product types, province, and frequency of cannabis use over time.

**Results:**

The percentage of consumers who sourced “all” their cannabis products from legal sources in the past 12 months varied by product type, ranging from 49% of solid concentrate consumers to 82% of cannabis drink consumers in 2021. The percentage of consumers sourcing “all” their respective products legally was greater in 2021 than 2020 across all products. Legal sourcing varied by frequency of use: weekly or more frequent consumers were more likely to source “some” (versus “none”) of their products legally versus less frequent consumers. Legal sourcing also varied by province, with a lower likelihood of legal sourcing in Québec of products whose legal sale was restricted (e.g., edibles).

**Conclusion:**

Legal sourcing increased over time, demonstrating progress in the transition to the legal market for all products in the first three years of legalization in Canada. Legal sourcing was highest for drinks and oils and lowest for solid concentrates and hash.

**Supplementary Information:**

The online version contains supplementary material available at 10.1186/s12954-023-00753-6.

## Introduction

In October 2018, Canada became the second country after Uruguay, to legalize and regulate the production, distribution, sale, possession and use of non-medical (‘recreational’) cannabis. The objectives of the Cannabis Act have a public health focus including to protect youth by restricting their access, to reduce the burden on the criminal justice system, to provide a quality-controlled legal supply of cannabis, and to create a regulated cannabis market and transition consumers from illegal to legal retail sources [1]. Transitioning consumers to the legal market is essential for ensuring the effectiveness of other regulatory measures intended to protect public health, including product standards, product labelling, and age verification checks. In the first year of legalization in Canada, only dried flower and some cannabis oils were available for legal purchase [2]. Retail sales from legal sources were estimated to account for 25% of the total dried flower market, with large variation across the provinces, ranging from 13% in Ontario to 70% in Prince Edward Island [3]. Other products, such as edibles, topicals, and extracts, were only made available from legal stores beginning December 2019 [2]. After the expansion of legal sales to include non-flower products, it is important to examine whether consumers are using the legal market to source all their edibles, topicals, and extracts, as well as dried flower.

Research from North America with legal non-medical markets demonstrates that consumer transition to the legal market is not immediate and the illegal market continues after years of legal access [4–10]. Data from the Canadian Cannabis Survey—Canada’s national monitoring survey for cannabis—suggests that the percentage of consumers sourcing from the legal market increased over the first three years after legalization (e.g., 25% usually sourcing from legal storefronts in 2019 to 53% in 2021), while the percentage of consumers sourcing cannabis from the illegal market decreased (e.g., 7% usually sourcing from illegal storefronts in 2019 to 2% in 2021) [6–8]. However, research to date has focused on legal purchases of either dried flower only or of all cannabis product types collectively. There is very little evidence on differences in legal sourcing across the range of different cannabis products, many of which are increasing in popularity of use [11].

Non-medical cannabis was regulated at the federal level under the Cannabis Act; however, provinces and territories have jurisdiction over retail policies. Indeed, legal cannabis markets vary across the Canadian provinces and territories, in that they could be treated as 13 distinct cannabis markets rather than one Canadian market. For example, the markets vary across provinces in areas such as retail structure (i.e., private only, government-run only, or a hybrid), minimum legal age (18 years in Alberta, 21 in Quebec, and 19 in all other provinces), access to retail stores (e.g., the number of stores ranged from four in Prince Edward Island to 1042 in Ontario in September 2021), price, and product standards [10, 12–16]. The extent to which restrictions on specific product types affect legal sourcing has yet to be examined. To date, Québec is the only Canadian province to have implemented regulations restricting THC content and product forms. Québec is Canada’s second most populated province, has a government-run retail system, and is the only province to restrict products available to purchase [17]. All cannabis products in Québec have a THC limit of 30%. While this limit does not restrict dried flower that has a biological ceiling of approximately 30% [18], it does restrict sales of high-potency concentrates [12–19]. Furthermore, the sale of edible cannabis products is not permitted in Québec if attractive to youth, including candy and chocolate [17]. Additionally, cannabis vaping oils are not sold in the Québec government-run retail stores, following a recommendation from Québec Public Health Director [20]. Research is needed on whether these additional product restrictions in Québec affect how many consumers purchase from the legal market. A greater understanding of how sales of varied cannabis products differ in Québec compared to other Canadian provinces will provide novel insight into the effects of different regulatory approaches to inform drug policy worldwide.

To our knowledge, this is the first study to examine legal sourcing of different cannabis products in the Canadian cannabis market. The aims of the study were to (1) examine the percentage of respondents sourcing all their cannabis products legally among past 12-month cannabis consumers of provincial legal age to purchase cannabis and (2) examine the association between legal sourcing, province, and cannabis use frequency in 2019–2021. We hypothesized that (1) the percentage of consumers sourcing all their respective cannabis products legally will increase over time, due to the expansion of the cannabis market in product offerings and size [2, 10, 15, 21, 22]; (2) legal sourcing of edibles will be lower in Québec than other provinces, due to their restriction of edibles that are attractive to youth [17]; and (3) frequent consumers of cannabis products will be less likely to source legally than occasional consumers, due to poorer perceptions of legal cannabis [22, 23], loyalty to dealers [23], or the fact that purchase limits in the legal market prevent large quantity discounts [12, 24–26].

## Methods

Data are from the International Cannabis Policy Study (ICPS), which consists of repeat cross-sectional surveys conducted annually in Canada. Data were collected via self-completed web-based surveys in September–October in 2019, 2020, and 2021 from respondents aged 16–65. A non-probability sample of respondents was recruited through the Nielsen Consumer Insights Global Panel and their partners’ panels. Nielsen draws stratified random samples from the online panels, with quotas based on age and province of residence. Nielsen emails panelists an invitation to access the survey via a hyperlink; respondents are unaware of the survey topic prior to accessing the link. Respondents confirm their eligibility and provide consent before completing the survey. Surveys were conducted in English or French. Median survey time was 25 min in 2019, 21 min in 2020, and 22 min in 2021. Upon completion, respondents received remuneration. The current study reported data from the Canadian sample. The survey had an American Association for the Public Opinion Research (AAPOR) cooperation rate of 63% in 2019, 66% in 2020, and 68% in 2021 [27].

The study was reviewed by and received ethics clearance through a University of Waterloo Research Ethics Committee (ORE#31,330). A full description of the study methods can be found in the ICPS Technical Reports and methodology paper [28–30].

## Measures

*Socio-demographic measures* were sex at birth, age, ethnicity/race, highest education level, perceived income adequacy, device type used to complete survey, and province of residence (Table 1). Both gender and sex at birth were collected separately in the International Cannabis Policy Study; however, sex at birth was included in the current study to retain all respondents (gender included missing data and very small cell sizes for non-cis individuals, which could not be included in analyses). For “perceived income adequacy” and “highest level of education”, those who answered, “Don’t know” or “Refuse to answer” were categorized to “Not stated”. Minimum legal age to purchase cannabis (MLA) was taken from provincial laws in September 2019, 2020, and 2021. For province of residence, Atlantic provinces (New Brunswick, Nova Scotia, Prince Edward Island, Newfoundland and Labrador) and Prairie provinces (Alberta, Saskatchewan, Manitoba) were combined due to small sample sizes in regression analysis and similarities in cannabis retail structure. Québec was chosen as the reference category in regression models due to its contrast to most provinces on product standards and retail structure.

**Table 1:** Unweighted and weighted sample characteristics of past 12-month cannabis consumers in Canada in 2019, 2020, and 2021 (*n* = 15,311)

	Unweighted % (*n*)			Weighted % (n)		
	2019 *n* = 4857	2020 *n* = 4652	2021 *n* = 5802	2019 *n* = 4867	2020 *n* = 4728	2021 *n* = 5716
*Age group*						
MLA-25	14.5 (702)	11.6 (540)	10.1 (584)	13.8 (672)	9.5 (450)	12.8 (732)
26–35	26.6 (1293)	24.4 (1133)	26.2 (1521)	30.5 (1482)	31.3 (1480)	29.9 (1707)
36–45	23.1 (1121)	22.2 (1033)	25.0 (1448)	22.5 (1094)	23.7 (1120)	24.9 (1422)
46–55	18.0 (876)	19.9 (921)	17.3 (1002)	18.4 (894)	19.0 (896)	17.2 (985)
56–65	17.8 (865)	22.0 (1025)	21.5 (1247)	14.9 (726)	16.5 (782)	15.2 (870)
*Sex at birth*						
Female	58.6 (2848)	60.7 (2822)	57.2 (3317)	45.5 (2213)	46.9 (2217)	47.0 (2686)
Male	41.4 (2009)	39.3 (1830)	42.8 (2485)	54.5 (2654)	53.1 (2512)	53.0 (3030)
*Race/Ethnicity*						
Black	3.1 (150)	2.5 (115)	3.2 (188)	4.0 (193)	3.8 (180)	3.6 (205)
East/Southeast Asian	4.6 (222)	4.6 (212)	5.6 (325)	4.7 (227)	4.9 (232)	5.0 (288)
Indigenous	3.9 (187)	3.1 (146)	3.2 (185)	4.0 (193)	2.7 (128)	3.6 (206)
Latinx	1.4 (66)	1.2 (56)	2.1 (123)	1.8 (85)	1.6 (76)	2.6 (150)
Middle Eastern	0.8 (40)	1.1 (53)	1.6 (94)	0.7 (36)	1.3 (61)	1.5 (85)
South Asian	2.2 (109)	2.4 (113)	2.8 (163)	2.7 (132)	3.0 (140)	3.1 (176)
White	77.2 (3749)	78.4 (3647)	74.2 (4304)	74.6 (3633)	76.0 (3594)	72.6 (4148)
Other/Mixed	6.9 (334)	6.7 (310)	7.2 (420)	7.6 (368)	6.7 (317)	8.0 (458)
*Education*						
Less than high school	6.2 (303)	6.0 (278)	5.8 (334)	12.1 (587)	9.4 (444)	12.2 (696)
High school diploma	17.4 (844)	16.6 (771)	15.9 (925)	28.0 (1361)	29.4 (1388)	29.0 (1656)
Some college or technical vocation	46.5 (2259)	44.8 (2086)	44.2 (2567)	36.1 (1758)	36.4 (1723)	35.8 (2049)
Bachelor’s degree or higher	28.9 (1404)	31.8 (1480)	33.4 (1935)	22.7 (1106)	24.0 (1133)	22.2 (1270)
Unstated	1.0 (47)	0.8 (37)	0.7 (41)	1.2 (56)	0.9 (41)	0.8 (45)
*Income adequacy*						
Very difficult	11.0 (532)	9.0 (417)	9.8 (566)	10.9 (533)	9.4 (442)	10.8 (615)
Difficult	24.4 (1183)	21.6 (1005)	20.8 (1209)	25.1 (1222)	20.9 (987)	22.0 (126)
Neither easy nor difficult	33.6 (1634)	36.4 (1692)	34.8 (2018)	33.5 (1628)	36.8 (1742)	34.6 (1980)
Easy	19.4 (944)	21.2 (985)	21.0 (1216)	18.6 (906)	21.2 (1003)	19.3 (1101)
Very Easy	9.2 (449)	9.7 (449)	11.5 (668)	8.8 (430)	9.2 (437)	10.3 (590)
Unstated	2.4 (115)	2.2 (104)	2.2 (125)	3.1 (148)	2.5 (118)	3.0 (174)
*Province of residence*						
British Columbia	15.3 (743)	16.8 (782)	14.0 (811)	14.5 (705)	15.1 (713)	15.4 (880)
Alberta	15.6 (757)	15.7 (729)	13.5 (781)	13.0 (633)	12.5 (590)	12.8 (730)
Saskatchewan	5.4 (260)	6.3 (292)	4.8 (279)	3.1 (150)	3.6 (168)	3.0 (174)
Manitoba	6.0 (289)	5.6 (258)	5.3 (308)	3.7 (180)	3.4 (162)	3.8 (220)
Ontario	23.4 (1134)	20.6 (959)	33.3 (1932)	41.1 (1998)	40.6 (1919)	40.6 (2322)
Québec	18.4 (892)	13.5 (626)	12.6 (729)	17.5 (853)	17.6 (830)	17.4 (994)
New Brunswick	5.1 (246)	7.1 (332)	5.6 (322)	2.3 (110)	2.4 (111)	2.3 (129)
Nova Scotia	6.8 (328)	7.1 (328)	6.1 (355)	3.1 (149)	3.0 (141)	2.9 (167)
Prince Edward Island	0.8 (41)	1.3 (62)	1.1 (65)	0.4 (20)	0.4 (19)	0.4 (22)
Newfoundland & Labrador	3.4 (167)	6.1 (284)	3.8 (220)	1.4 (70)	1.6 (75)	1.4 (79)

Income adequacy is assessed by the question: “Thinking about your family’s income, how difficult or easy is it to make ends meet?”, where ‘making ends meet’ means having enough money to pay for the things your family needs

*Cannabis product use frequency*: Past 12-month cannabis consumers were asked whether they used each of ten product types in the past 12 months (No; Yes, but not in past 12 months; Yes, in past 12 months): dried flower (smoked or vaped), cannabis oils/liquids taken orally (drops or capsules), cannabis oil/liquid for vaping, edibles/foods, drinks, solid concentrates (e.g., wax, shatter), hash or kief, tinctures, and topicals. Respondents who reported using a specific product in the past 12 months were asked to provide their frequency of using each product: “Less than monthly, but in the past year”, “Monthly”, “Weekly”, “Daily/almost daily”, “Don’t know”. For regression analysis, responses were dichotomized to “Occasional consumer” (Less than monthly/Monthly) and “Frequent consumer” (Weekly/Daily or almost daily) due to small sample sizes.

*Legal sourcing of cannabis products in the past 12 months*: Past 12-month cannabis consumers of each cannabis product were asked “Overall, about what percentage (%) of the [products] that you used in the past 12 months came from LEGAL/AUTHORIZED sources?” Respondents could enter a numerical value between 0 and 100. Although this variable represents a continuous outcome, the distribution did not follow a normal distribution due to a high number of responses at either extreme (0% and 100%). As a result, responses were categorized into: “All” (100%), “Some” (1–99%), and “None” (0%). Previous analyses examined different classification schemes for this outcome, but due to a high level of clustering at the extremes, a five-level outcome and a continuous outcome were rejected for the current three-level outcome [24].

All questions included “Don’t know” and “Refuse to answer” options and were excluded unless specified within the measures above.

### Statistical analysis

The final Canadian cross-sectional samples comprised 15,256 respondents in 2019, 15,780 in 2020, and 16,952 in 2021. Analyses were conducted on the sub-sample of respondents who had consumed cannabis in the past 12 months and were of provincial legal age to purchase legal cannabis products (*n*_2019_ = 4857; *n*_2020_ = 4652; *n*_2021_ = 5802). Missing data were removed using case-wise deletion for variables used in regression models: highest level of education (*n* = 125); perceived income adequacy (*n* = 344); and frequency of product use (dried flower: *n* = 17; oral oils: *n* = 3; capsules: *n* = 7; vape oils: *n* = 28; edibles: *n* = 34; drinks: *n* = 34; solid concentrates: *n* = 45; hash/kief: *n* = 48; tinctures: *n* = 49; topicals: *n* = 39). Respondents who did not provide a percentage to the outcome measure were removed in regression models; sensitivity analyses were conducted with and without these respondents and interpretations remained consistent.

Post-stratification sample weights were constructed based on the Canadian Census estimates. In 2019, 2020, and 2021, respondents were classified into age-by-sex-by-province, education, and age-by-tobacco smoking status groups. A raking algorithm was applied to the cross-sectional analytic samples to compute weights that were calibrated to these groupings. Weights were rescaled to the sample size for all years in Canada. Estimates are weighted unless otherwise specified.

First, descriptive statistics were used to describe the percentage of respondents who reported sourcing “all”, “some”, or “none” of their respective cannabis products legally in each year. Second, ten multinomial logistic regression models, one for each product, were fitted to estimate the association between legal sourcing, province of residence and product use frequency over time. In all models, two-way interactions between survey year and product use frequency were tested. All models were adjusted for age, sex at birth, education level, ethnicity/race, income adequacy, and survey device type. Adjusted odds ratios (AORs) are reported with 95% confidence intervals (95% CI). Analyses were conducted using survey procedures in SAS (SAS version 9.4, SAS Institute Inc., Cary, NC, USA).

## Results

Table 1 displays the weighted and unweighted sample characteristics of Canadian past 12-month cannabis consumers of provincial legal age, 2019–2021. Across all years, close to half of respondents were male, three-quarters identified as white ethnicity/race, and one quarter were educated to a bachelor’s degree or higher.

**Fig. 1:**
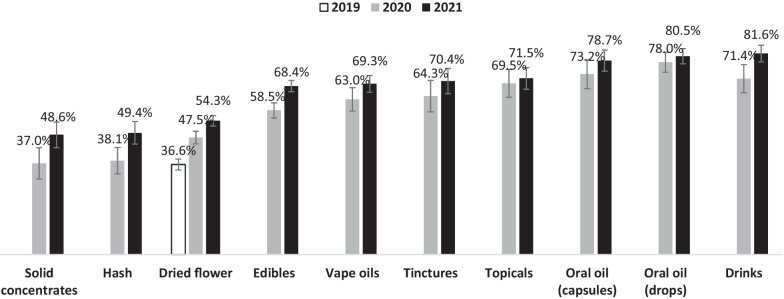
**Percentage of respondents who sourced “ALL” of their respective products from legal sources in the past 12 months, 2019–2021.** Denominator is among respondents who consumed the respective cannabis products in the past 12 months and provided an answer to the sourcing question. Only dried flower was legal for purchase in 2019. Sample sizes for each product category were: solid concentrates: *n* = 906; Hash: *n* = 1,253; Tinctures: *n* = 897; Topicals: *n* = 1160; Dried flower: *n* = 8432; Vape oils: *n* = 1,840; Edibles: *n* = 4137; Drinks: *n* = 1316; Oral oil (capsules): *n* = 998; Oral oil (drops): *n* = 1989

Figure 1 displays the percentage of respondents who sourced “all” their respective cannabis product type from legal sources in past 12 months. In 2021, the percentage of consumers who sourced “all” of their respective cannabis product type legally ranged from 48.6% of solid concentrate consumers to 81.6% of cannabis drink consumers. Across all products, the percentage of consumers sourcing “all” their respective products legally were greater in 2021 versus 2020.

Additional file 1 and Additional file 2 display the percentage of cannabis product consumers who sourced “some” and “none” of their respective products legally in past 12 months. The percentage of consumers who sourced “some” of their respective cannabis products legally ranged from 12.8% of oral oil (drop) consumers to 36.4% of dried flower consumers in 2021. The percentage of consumers who sourced “none” of their respective cannabis products legally ranged from 4.4% of capsule consumers to 31.4% of hash/kief consumers in 2021.

### Trends in legal sourcing across years

Table 2 and Additional file 3, Additional file 4 and Additional file 5 display the multinomial logistic regression analyses of legal sourcing of cannabis products. Survey year was associated with increased legal sourcing of six cannabis products: consumers of dried flower, solid concentrates, vape oils, edibles, hash/kief, and drinks had higher odds of sourcing “all” of their respective product legally in 2021 versus 2020. In 2021, consumers of dried flower, edibles and hash/kief had higher odds of sourcing “some” of their respective product legally versus 2020.

**Table 2:** Weighted multinomial logistic regression analysis for dried flower sourced from legal sources in the past 12 months among cannabis consumers of legal age to purchase cannabis, 2019–2021 (*n* = 8295)

	Some (vs. None)	All (vs. None)
	AOR (95% CI)	AOR (95% CI)
*Survey year*		
2020 (vs. 2019)	**1.56 (1.27, 1.93)**	**2.26 (1.84, 2.77)**
2021 (vs. 2019)	**2.25 (1.83, 2.76)**	**4.10 (3.35, 5.00)**
2021 (vs. 2020)		
*Dried flower frequency*		
Frequent	**3.01 (2.52, 3.60)**	0.93 (0.79, 1.10)
Occasional	REF	REF
*Province of residence*		
Québec	REF	REF
British Columbia	**0.70 (0.53, 0.93)**	**0.53 (0.40, 0.68)**
Prairie provinces	1.17 (0.89, 1.53)	1.08 (0.84, 1.39)
Ontario	0.82 (0.64, 1.06)	**0.58 (0.45, 0.74)**
Atlantic provinces	**1.62 (1.20, 2.20)**	**1.51 (1.14, 2.01)**
*Age*		
MLA-25	**4.00 (2.81, 5.70)**	**3.88 (2.76, 5.45)**
26–35	**2.74 (2.11, 3.55)**	**2.73 (2.13, 3.49)**
36–45	**1.87 (1.45, 2.42)**	**1.98 (1.56, 2.52)**
46–55	**1.39 (1.07, 1.81)**	**1.35 (1.06, 1.73)**
56–65	REF	REF
*Sex at birth*		
Female	REF	REF
Male	**1.25 (1.05, 1.49)**	1.14 (0.97, 1.35)
*Ethnicity/Race*		
Mixed/Other	**1.29 (1.03, 1.61)**	0.95 (0.77, 1.18)
White	REF	REF
*Highest level of Education*		
Less than high school	REF	REF
High school diploma	1.38 (0.99, 1.92)	**1.73 (1.24, 2.42)**
Some college or technical vocation	1.29 (0.95, 1.75)	**1.73 (1.28, 2.35)**
Bachelor’s degree or higher	**1.78 (1.27, 2.51)**	**2.85 (2.03, 3.99)**
*Income adequacy*		
Very difficult/Difficult	REF	REF
Neither easy nor difficult	0.92 (0.75, 1.14)	1.13 (0.92, 1.37)
Easy/Very easy	**0.77 (0.62, 0.96)**	1.06 (0.86, 1.31)
*Survey device*		
Smartphone	**1.27 (1.06, 1.53)**	1.00 (0.84, 1.19)
Tablet	0.86 (0.58, 1.28)	1.07 (0.73, 1.56)
Computer	REF	REF

Bold indicates significance at *p* < 0.05

### Differences in legal sourcing by frequency of use

Product frequency of use was associated with legal sourcing of all cannabis products except solid concentrates (Table 2, Additional file 3, Additional file 4 and Additional file 5). Frequent consumers had higher odds of sourcing “some” of their product legally versus occasional consumers.

An overall interaction between survey wave and frequency of product use was significant for three cannabis products: dried flower (*F* = 5.07, *p* < 0.001), oral oils (*F* = 6.81, *p* = 0.001), and tinctures (*F* = 6.73, *p* = 0.001). Frequent dried flower consumers had higher odds of sourcing “all” or “some” of their dried flower legally in 2021 versus 2020 and 2019, and in 2020 versus 2019. Occasional dried flower consumers had higher odds of sourcing “all” or “some” of their dried flower legally in 2021 versus 2020 and 2019, and “all” of their dried flower in 2020 versus 2019. Frequent consumers of oral oils and tinctures had higher odds of sourcing “all” or “some” of their oral oils or tinctures legally in 2021 versus 2020.

### Differences in legal sourcing by province

Province of residence was associated with legal sourcing of five cannabis products: dried flower, vape oils, edibles, oral oils, and tinctures (Table 2, Additional file 3, Additional file 4 and Additional file 5). Dried flower consumers in Québec had higher odds of sourcing “all” their dried flower legally versus British Columbia and Ontario. Compared to dried flower consumers in Québec, consumers in the Atlantic provinces had higher odds of sourcing “all” or “some” of their dried flower legally. Oral oil consumers in Québec had higher odds of sourcing “some” of their oral oils legally versus those in all provinces except Ontario. Compared to vape oil consumers in Québec, consumers in the Prairie provinces and Ontario had higher odds of sourcing “all” of vape oils legally. Compared to edible consumers in Québec, consumers in all provinces had higher odds of sourcing “all” their edibles legally. Tincture consumers in Québec had higher odds of sourcing “some” of their tinctures legally versus the Prairie provinces. Compared to tincture consumers in Québec, consumers in Ontario had higher odds of sourcing “all” their tinctures legally.

### Sociodemographic differences in legal sourcing

After adjusting for covariates, age was associated with legal sourcing of all cannabis products, where younger respondents had higher odds of sourcing at least some product legally (Table 2, Additional file 3, Additional file 4 and Additional file 5). Sex at birth was associated with legal sourcing of three cannabis products: dried flower, cannabis drinks (male respondents had higher odds of sourcing “some”) and tinctures (female respondents had higher odds of sourcing “all”). Ethnicity/race was associated with legal sourcing of all cannabis products except cannabis drinks and vape oils, where respondents identifying as mixed/other ethnicity/race had higher odds of sourcing “some” of their products legally. Education level was associated with legal sourcing of all cannabis products except cannabis drinks, where respondents with a bachelor’s degree had higher odds of sourcing at least some product legally. Income adequacy was associated with legal sourcing of six cannabis products: dried flower, edibles, topicals, tinctures, oral oils, and capsules.

## Discussion

The percentage of Canadian cannabis consumers who sourced their products from legal sources in the three years after legalization varied across cannabis products, ranging from approximately half of solid concentrate consumers sourcing all their product legally to 82% of cannabis drink consumers. High percentages of cannabis drink consumers sourcing legally could be explained by drinks being a ‘newer’ product category for the legal market that wasn’t as accessible in the illegal markets [26]. Products typically perceived as ‘medical’ products, such as oral oils and capsules, had greater percentages of consumers sourcing all their product legally. Oral oils were available legally for medical purposes prior to 2018; therefore, consumers may have already been sourcing legally through licensed producers before non-medical legalization [31]. Moreover, consumers sourcing ‘medical’ products may prioritize legal products where the quality is regulated, tested, and standardized to better dose their medication. In contrast, solid concentrates and hash products had the lowest percentage of consumers sourcing legally, suggesting the greatest competition between the illegal and legal market is in these products. This may be due to more established consumers having closer contacts with dealers for these products as well as retail stores not stocking these products until later in our study period. Indeed, solid concentrates and hash were reportedly only entering retail stores around April/May 2020 [32].

Different studies have used varied metrics for estimating legal sourcing or purchases of cannabis in Canada. For example, some studies report “overall” sourcing, whereas others report “last purchase”, as well as representing legal sourcing as an overall percentage versus categories (“any” or “all”) [6–8, 10, 33]. These produce different point estimates, but the trends across these different measures are highly consistent in showing that legal sourcing/purchasing has increased, and illegal sourcing/purchasing has decreased since legalization. These trends are also seen in the current study, where legal sourcing of six cannabis products was more likely in 2021 than 2020.

Consumers of six out of 10 cannabis products were more likely to source all their respective products legally in 2021 versus 2020, suggesting that the legal market is becoming more competitive across some types of products. These results mirror the general trend demonstrated by the Canadian Cannabis Survey, whereby consumers are increasingly sourcing all cannabis from the legal market [6–8]. Indeed, the Canadian government has documented increasing legal sales [34]. A study examining Canadian cannabis sales during the COVID-19 pandemic demonstrated that the increase in cannabis sales during this time were more attributable to the expansion of cannabis products in January 2020, rather than to the pandemic [21]. This suggests that consumers were entering the legal market for non-flower products so we may expect the percentage of those sourcing legally to increase in coming years.

We hypothesized that frequent consumers would be less likely than occasional consumers to source legally due to poorer perceptions of legal cannabis [22, 23], loyalty to dealers [23], or the fact that purchase limits in the legal market prevent large quantity discounts [12, 24–26]. However, frequent consumers were more likely to source *some* of their products legally and were equally likely to source *all* their respective products legally as occasional consumers. This is particularly important given that frequent consumers consume approximately 80% of all cannabis consumed [35–37], and therefore are an important group to encourage to use the legal market. These findings suggest that frequent consumers, when defined as weekly or daily consumers, are transitioning to the legal market, which was one of the key public health objectives of the Cannabis Act.

The legal cannabis market in Canada differs between provinces in areas such as retail structure, minimum legal age, product standards [13, 14], access to retail stores [15], prices [10, 12], and retail sales [16]. Provincial differences were observed in the sourcing of dried flower, oral oil, vape oils, tinctures, and edibles. Edibles consumers in all provinces were more likely to source all their edibles legally than those in Québec. Québec is the only province to restrict edible products that may appeal to youth and therefore removes the most popular edible forms: candy and chocolate. At the time of writing—August 2022—there were only seven edible products available for purchase from the Québec online store compared to 262 products on the Ontario online store [38, 39]. Québec edibles consumers may not source their edibles legally because their legal options are limited. They may legally make their own edibles at home, but this may not be as convenient as using the illegal market. Home cultivation is also prohibited in Québec, further limiting opportunities to make homemade edibles. Further research is needed to understand whether these restrictions in legal edibles in Québec results in illegal sourcing, homemade edibles, or shifting consumption to other cannabis products that are available to purchase. The lack of regulation in homemade edibles or edibles sourced illegally may be a public safety concern due to inconsistent dosing in products and potential overconsumption.

In contrast, dried flower consumers in Québec were more likely to source all their dried flower legally than those in British Columbia and Ontario, and less likely to do so than those in the Atlantic provinces. Québec has had some of the lowest legal dried flower prices since legalization, making legal dried flower potentially more desirable than illegal dried flower in that province compared to other provinces [10, 12, 26, 40]. Further research should examine how consumption of these products varies across the provinces, and whether substitutions occur between cannabis products in response to price and availability from legal sources.

### Limitations

This study is subject to limitations. Respondents were recruited using non-probability-based sampling; therefore, the findings do not necessarily provide nationally representative estimates. The data were weighted by age group, sex, region, education, and tobacco smoking status in Canada. Cannabis use estimates were generally lower than national estimates for young adults, and higher than national surveys in Canada. This is likely because the ICPS sampled individuals aged 16–65, whereas national surveys included older adults, who are known to have lower rates of cannabis use.

There are limitations in the classification used for the outcome variable. While “all” and “none” are definitive categories (100% and 0%), the “some” category is broader as it contains all respondents between 1 and 99%. Previous work examined alternative classifications, including a five-level outcome and a continuous outcome [41]. A comparison of model performance indicated that multinomial models had substantially lower AIC values versus the linear model, suggesting a better overall fit with the data. R^2^ values were similar between the multinomial models, with substantially lower AIC values for the 3-level multinomial model. In addition, the focus of the paper was those sourcing “all” their products legally, which was a more clear, unambiguous outcome.

The COVID-19 pandemic may have impacted how consumers accessed their cannabis products by decreasing access to legal sources for a short period of time. In Canada, the pandemic also coincided with market expansion and the introduction of edibles, extracts, and topical products into the market from December 2019 [21]. The release of edibles, extracts, and topical cannabis products was staggered with some products unavailable to purchase until May 2020 [32]. Therefore, lower rates of legal sourcing may depend on what products were available in legal stores.

## Conclusions

Legal sourcing of cannabis was greater in 2021 than 2020 for all ten cannabis products. In 2021, the percentage of consumers sourcing all their products legally in the past 12 months ranged from 49% of solid concentrate consumers in 2021 to 82% of cannabis drink consumers. Meanwhile, the percentage that sourced “none” was lower in 2021 than 2020 for all cannabis products. Legal purchasing varied across the provinces, including for products such as edibles and vapes, that were restricted for legal sale in Québec. Transitioning consumers of all cannabis products into the regulated market is important for public health and safety. Future studies should continue to examine cannabis product sourcing in Canada over time, as well as ways to displace the illegal market for all cannabis products without also promoting the use of high-potency cannabis products.

## Supplementary Information

**Additional file 1**. Percentage respondents who sourced “SOME” their respective products from legal sources in the past 12 months, 2019-2021.

**Additional file 2**. Percentage respondents who sourced “NONE” their respective products from legal sources in the past 12 months, 2019-2021.

**Additional file 3**. Weighted multinomial logistic regression analysis for products sourced from legal sources in the past 12 months among cannabis consumers of legal age to purchase cannabis, 2019-2021.

**Additional file 4**. Weighted multinomial logistic regression analysis for products sourced from legal sources in the past 12 months among cannabis consumers of legal age to purchase cannabis, 2019-2021.

**Additional file 5**. Weighted multinomial logistic regression analysis for products sourced from legal sources in the past 12 months among cannabis consumers of legal age to purchase cannabis, 2019-2021.

## Data Availability

The data that support the findings of this study are available from the International Cannabis Policy Study (www.cannabisproject.ca) but restrictions apply to the availability of these data, and so are not publicly available. Data are, however, available from the authors upon reasonable request.
