# Supplementary material for: Legal sourcing of ten cannabis products in the Canadian cannabis market, 2019–2021: a repeat cross-sectional study
Source: Harm Reduct J. 2023 Feb 17;20:19. doi: 10.1186/s12954-023-00753-6 (PMC9936931; doi:10.1186/s12954-023-00753-6)
Supplement: Supplementary file 1 — Additional file 1. Percentage respondents who sourced “SOME” their respective products from legal sources in the past 12 months, 2019-2021. [file 12954_2023_753_MOESM1_ESM.docx]

**Additional File 1: Percentage respondents who sourced “SOME” their respective products from legal sources in the past 12 months, 2019-2021**

Denominator is among respondents who consumed the respective cannabis products in the past 12 months and provided an answer to the sourcing question. Sample sizes for each product category were: solid concentrates: n=906; Hash: n=1,253; Tinctures: n=897; Topicals: n=1,160; Dried flower: n=8,432; Vape oils: n=1,840; Edibles: n=4,137; Drinks: n=1,316; Oral oil (capsules): n=998; Oral oil (drops): n=1,989.
