# Supplementary material for: Legal sourcing of ten cannabis products in the Canadian cannabis market, 2019–2021: a repeat cross-sectional study
Source: Harm Reduct J. 2023 Feb 17;20:19. doi: 10.1186/s12954-023-00753-6 (PMC9936931; doi:10.1186/s12954-023-00753-6)
Supplement: Supplementary file 3 — Additional file 3. Weighted multinomial logistic regression analysis for products sourced from legal sources in the past 12 months among cannabis consumers of legal age to purchase cannabis, 2019-2021. [file 12954_2023_753_MOESM3_ESM.docx]

**Additional File 3 – Weighted multinomial logistic regression analysis for products sourced from legal sources in the past 12 months among cannabis consumers of legal age to purchase cannabis, 2019-2021**

|  | **Solid concentrates**  n=888 | | **Vape oils**  n=1,806 | | **Edibles**  n=4,083 | |
| --- | --- | --- | --- | --- | --- | --- |
|  | **Some**  (vs. None) | **All**  (vs. None) | **Some**  (vs. None) | **All**  (vs. None) | **Some**  (vs. None) | **All**  (vs. None) |
|  | **AOR (95% CI)** | **AOR (95% CI)** | **AOR (95% CI)** | **AOR (95% CI)** | **AOR (95% CI)** | **AOR (95% CI)** |
| **Survey year** |  |  |  |  |  |  |
| 2020 | REF | REF | REF | REF | REF | REF |
| 2021 | 1.32 (0.80, 2.17) | **1.77 (1.18, 2.66)** | 1.16 (0.74, 1.81) | **1.50 (1.04, 2.17)** | **1.49 (1.13, 1.96)** | **1.88 (1.52, 2.33)** |
| **Product use frequency** |  |  |  |  |  |  |
| Occasional | REF | REF | REF | REF | REF | REF |
| Frequent | 1.54 (0.90, 2.62) | 0.83 (0.52, 1.33) | **2.11 (1.31, 3.39)** | 1.31 (0.87, 1.96) | **2.10 (1.50, 2.93)** | 1.01 (0.75, 1.34) |
| **Province of residence** |  |  |  |  |  |  |
| Québec | REF | REF | REF | REF | REF | REF |
| British Columbia | 0.40 (0.14, 1.13) | 0.99 (0.43, 2.30) | 0.45 (0.20, 1.02) | 1.40 (0.69, 2.84) | 1.04 (0.60, 1.81) | **1.77 (1.16, 2.72)** |
| Prairie provinces | 0.66 (0.25, 1.74) | 1.43 (0.66, 3.09) | 0.70 (0.33, 1.51) | **2.46 (1.25, 4.82)** | 1.07 (0.63, 1.83) | **2.37 (1.59, 3.53)** |
| Ontario | 0.92 (0.36, 2.34) | 1.48 (0.67, 3.23) | 1.16 (0.56, 2.42) | **2.23 (1.14, 4.36)** | 1.27 (0.76, 2.10) | **1.66 (1.13, 2.43)** |
| Atlantic provinces | 0.97 (0.35, 2.67) | 1.64 (0.71, 3.80) | 0.59 (0.26, 1.31) | 1.24 (0.62, 2.47) | 1.48 (0.89, 2.52) | **1.88 (1.25, 2.81)** |
| **Age** |  |  |  |  |  |  |
| MLA-25 | **3.07 (1.12, 8.42)** | **3.43 (1.51, 7.78)** | **4.75 (1.80, 12.53)** | **3.12 (1.39, 7.01)** | 1.18 (0.70, 1.98) | **1.51 (1.01, 2.27)** |
| 26-35 | 2.12 (0.89, 5.03) | **2.30 (1.15, 4.61)** | **2.98 (1.37, 6.49)** | 1.69 (0.94, 3.05) | 1.55 (1.01, 2.38) | **1.42 (1.02, 1.98)** |
| 36-45 | 1.53 (0.61, 3.82) | **2.31 (1.14, 4.70)** | **2.45 (1.12, 5.38)** | 1.48 (0.81, 2.69) | 1.19 (0.78, 1.82) | 1.17 (0.85, 1.61) |
| 46-55 | 2.38 (0.89, 6.36) | **2.33 (1.10, 4.92)** | 1.92 (0.81, 4.51) | 1.36 (0.72, 2.58) | 1.38 (0.90, 2.14) | **1.55 (1.10, 2.17)** |
| 56-65 | REF | REF | REF | REF | REF | REF |
| **Sex at birth** |  |  |  |  |  |  |
| Female | REF | REF | REF | REF | REF | REF |
| Male | 1.21 (0.71, 2.04) | 1.22 (0.81, 1.84) | 1.37 (0.88, 2.13) | 0.87 (0.60, 1.24**)** | 1.07 (0.81, 1.42) | 0.94 (0.76, 1.17) |
| **Ethnicity/Race** |  |  |  |  |  |  |
| Mixed/Other | **2.55 (1.48, 4.41)** | 0.93 (0.57, 1.53) | 1.74 (1.06, 2.87) | 0.87 (0.56, 1.34) | **2.11 (1.51, 2.96)** | 1.20 (0.90, 1.58) |
| White | REF | REF | REF | REF | REF | REF |
| **Highest level of Education** |  |  |  |  |  |  |
| Less than high school | REF | REF | REF | REF | REF | REF |
| High school diploma | 1.46 (0.61, 3.50) | 1.71 (0.84, 3.50) | 0.98 (0.42, 2.31) | 1.76 (0.84, 3.65) | 0.97 (0.53, 1.79) | 1.12 (0.67, 1.89) |
| Some college or technical vocation | 1.15 (0.50, 2.65) | 1.64 (0.83, 3.22) | 0.61 (0.28, 1.32) | 1.25 (0.66, 2.39) | 0.71 (0.40, 1.25) | 1.11 (0.68, 1.80) |
| Bachelor’s degree or higher | **5.98 (2.38, 15.00)** | **3.42 (1.63, 7.66)** | 1.19 (0.52, 2.73) | **2.40 (1.17, 4.92)** | 1.10 (0.60, 2.00) | **1.91 (1.15, 3.20)** |
| **Income adequacy** |  |  |  |  |  |  |
| Very difficult/Difficult | REF | REF | REF | REF | REF | REF |
| Neither easy nor difficult | 0.78 (0.42, 1.43) | 1.14 (0.72, 1.81) | 1.07 (0.63, 1.85) | 1.30 (0.84, 2.02) | 1.06 (0.76, 1.48) | 1.14 (0.88, 1.48) |
| Easy/Very easy | 1.31 (0.68, 2.51) | 1.30 (0.76, 2.23) | 1.10 (0.64, 1.92) | 1.07 (0.68, 1.69) | 1.05 (0.73, 1.51) | **1.38 (1.04, 1.83)** |
| **Survey device** |  |  |  |  |  |  |
| Smartphone | 1.16 (0.67, 2.00) | 1.04 (0.67, 1.62) | 0.70 (0.43, 1.13) | 0.79 (0.53, 1.19) | 1.07 (0.80, 1.44) | 1.11 (0.88, 1.40) |
| Tablet | 0.51 (0.10, 2.73) | 0.73 (0.28, 1.96) | 0.36 (0.11, 1.13) | 0.73 (0.29, 1.88) | 0.69 (0.35, 1.38) | 0.61 (0.36, 1.02) |
| Computer | REF | REF | REF | REF | REF | REF |

Bolded values indicate significance at p<0.05.
