# Supplementary material for: Legal sourcing of ten cannabis products in the Canadian cannabis market, 2019–2021: a repeat cross-sectional study
Source: Harm Reduct J. 2023 Feb 17;20:19. doi: 10.1186/s12954-023-00753-6 (PMC9936931; doi:10.1186/s12954-023-00753-6)
Supplement: Supplementary file 4 — Additional file 4. Weighted multinomial logistic regression analysis for products sourced from legal sources in the past 12 months among cannabis consumers of legal age to purchase cannabis, 2019-2021. [file 12954_2023_753_MOESM4_ESM.docx]

**Additional File 4 – Weighted multinomial logistic regression analysis for products sourced from legal sources in the past 12 months among cannabis consumers of legal age to purchase cannabis, 2019-2021**

|  | **Oral oils: drops**  n=1,966 | | **Tinctures**  **n=881** | | **Oral oils: capsules**  n=978 | |
| --- | --- | --- | --- | --- | --- | --- |
|  | **Some**  (vs. None) | **All**  (vs. None) | **Some**  (vs. None) | **All**  (vs. None) | **Some**  (vs. None) | **All**  (vs. None) |
|  | **AOR (95% CI)** | **AOR (95% CI)** | **AOR (95% CI)** | **AOR (95% CI)** | **AOR (95% CI)** | **AOR (95% CI)** |
| **Survey year** |  |  |  |  |  |  |
| 2020 | REF | REF | REF | REF | REF | REF |
| 2021 | 1.05 (0.59, 1.86) | 1.36 (0.87, 2.11) | 1.41 (0.71, 2.79) | 1.51 (0.92, 2.49) | 1.56 (0.70, 3.45) | 1.94 (0.97, 3.88) |
| **Product frequency** |  |  |  |  |  |  |
| Frequent | **2.60 (1.41, 4.79)** | 1.23 (0.75, 2.00) | **2.37 (1.11, 5.06)** | 1.15 (0.64, 2.06) | **4.79 (1.63, 14.03)** | 2.12 (0.79, 5.64) |
| Occasional | REF | REF | REF | REF | REF | REF |
| **Province of residence** |  |  |  |  |  |  |
| Québec | REF | REF | REF | REF | REF | REF |
| British Columbia | **0.21 (0.06, 0.70)** | 0.53 (0.20, 1.42) | 0.32 (0.09, 1.18) | 1.26 (0.52, 3.11) | 0.39 (0.07, 2.31) | 0.61 (0.12, 3.11) |
| Prairie provinces | **0.20 (0.06, 0.66)** | 0.67 (0.26, 1.71) | 0.34 (0.10, 1.18) | 1.30 (0.53, 3.16) | 0.30 (0.06, 1.64) | 0.86 (0.18, 4.21) |
| Ontario | 0.80 (0.26, 2.44) | 0.90 (0.34, 2.40) | 1.09 (0.34, 3.48) | **2.51 (1.01, 6.23)** | 0.70 (0.14, 3.49) | 0.61 (0.13, 2.82) |
| Atlantic provinces | **0.24 (0.07, 0.85)** | 0.43 (0.16, 1.16) | 1.11 (0.28, 4.38) | 1.58 (0.58, 4.30) | 0.33 (0.05, 1.96) | 0.77 (0.16, 3.74) |
| **Age** |  |  |  |  |  |  |
| MLA-25 | **3.22 (1.10, 9.44)** | 1.34 (0.59, 3.02) | **6.58 (1.42, 30.49)** | 1.76 (0.57, 5.51) | **14.68 (2.93, 73.65)** | 3.38 (0.88, 12.99) |
| 26-35 | 2.21 (0.89, 5.48) | 1.02 (0.51, 2.03) | **6.33 (2.04, 19.68)** | 1.26 (0.67, 2.80) | **6.24 (1.62, 24.07)** | 2.29 (0.73, 7.20) |
| 36-45 | 1.96 (0.76, 5.07) | 0.99 (0.49, 2.03) | 1.69 (0.55, 5.17) | 0.57 (0.28, 1.17) | **5.75 (1.51, 21.91)** | 1.92 (0.62, 5.99) |
| 46-55 | 0.80 (0.31, 2.02) | 0.87 (0.46, 1.63) | 1.70 (0.54, 5.41) | 0.96 (0.45, 2.04) | 1.55 (0.34, 7.07) | 1.49 (0.43, 5.06) |
| 56-65 | REF | REF | REF | REF | REF | REF |
| **Sex at birth** |  |  |  |  |  |  |
| Female | REF | REF | REF | REF | REF | REF |
| Male | 1.20 (0.67, 2.13) | 0.74 (0.47, 1.17) | 1.19 (0.59, 2.41) | **0.48 (0.29, 0.80)** | 1.24 (0.52, 2.96) | 1.00 (0.47, 2.17) |
| **Ethnicity/Race** |  |  |  |  |  |  |
| Mixed/Other | **4.70 (2.21, 9.99)** | 1.64 (0.85, 3.15) | **2.80 (1.26, 6.23)** | 1.14 (0.60, 2.17) | **2.93 (1.04, 8.31)** | 1.44 (0.55, 3.75) |
| White | REF | REF | REF | REF | REF | REF |
| **Highest level of Education** |  |  |  |  |  |  |
| Less than high school | REF | REF | REF | REF | REF | REF |
| High school diploma | 0.99 (0.62, 3.15) | 1.41 (0.57, 3.48) | 0.61 (0.16, 2.25) | 0.96 (0.32, 2.84) | **6.30 (1.00, 39.56)** | 3.86 (0.77, 19.31) |
| Some college or technical vocation | 0.60 (0.22, 1.68) | 1.69 (0.76, 3.74) | 0.35 (0.10, 1.24) | 1.38 (0.50, 3.77) | 2.36 (0.49, 11.32) | 2.86 (0.80, 10.23) |
| Bachelor’s degree or higher | 1.85 (0.61, 5.63) | **3.07 (1.24, 7.61)** | 1.20 (0.33, 4.40) | 2.37 (0.79, 7.07) | 4.87 (0.89, 26.65) | 3.19 (0.76, 13.33) |
| **Income adequacy** |  |  |  |  |  |  |
| Very difficult/Difficult | REF | REF | REF | REF | REF | REF |
| Neither easy nor difficult | 1.35 (0.67, 2.73) | 1.65 (0.95, 2.86) | 0.99 (0.39, 2.52) | 1.74 (0.95, 3.17) | 2.39 (0.90, 6.39) | **2.69 (1.15, 6.30)** |
| Easy/Very easy | 1.40 (0.68, 2.87) | **2.24 (1.29, 3.89)** | **2.35 (1.02, 5.40)** | **2.40 (1.26, 4.56)** | 2.26 (0.71, 7.18) | 2.35 (0.85, 6.46) |
| **Survey device** |  |  |  |  |  |  |
| Smartphone | 1.02 (0.55, 1.90) | 0.99 (0.62, 1.61) | 1.06 (0.52, 2.16) | 1.40 (0.82, 2.37) | 0.34 (0.13, 0.86) | 0.42 (0.18, 0.96) |
| Tablet | 0.78 (0.19, 3.25) | 1.00 (0.32, 3.13) | **0.05 (0.01, 0.66)** | 0.59 (0.17, 2.09) | 0.37 (0.06, 2.42) | 0.43 (0.11, 1.76) |
| Computer | REF | REF | REF | REF | REF | REF |

Bolded values indicate significance at p<0.05.
