# Supplementary material for: Legal sourcing of ten cannabis products in the Canadian cannabis market, 2019–2021: a repeat cross-sectional study
Source: Harm Reduct J. 2023 Feb 17;20:19. doi: 10.1186/s12954-023-00753-6 (PMC9936931; doi:10.1186/s12954-023-00753-6)
Supplement: Supplementary file 5 — Additional file 5. Weighted multinomial logistic regression analysis for products sourced from legal sources in the past 12 months among cannabis consumers of legal age to purchase cannabis, 2019-2021. [file 12954_2023_753_MOESM5_ESM.docx]

**Additional File 5 – Weighted multinomial logistic regression analysis for products sourced from legal sources in the past 12 months among cannabis consumers of legal age to purchase cannabis, 2019-2021**

|  | **Topicals**  **n=1,149** | | **Hash/Kief**  **n=1,240** | | **Cannabis drinks**  n=1,291 | |
| --- | --- | --- | --- | --- | --- | --- |
|  | **Some**  (vs. None) | **All**  (vs. None) | **Some**  (vs. None) | **All**  (vs. None) | **Some**  (vs. None) | **All**  (vs. None) |
|  | **AOR (95% CI)** | **AOR (95% CI)** | **AOR (95% CI)** | **AOR (95% CI)** | **AOR (95% CI)** | **AOR (95% CI)** |
| **Survey year** |  |  |  |  |  |  |
| 2020 | REF | REF | REF | REF | REF | REF |
| 2021 | 1.44 (0.76, 2.72) | 1.35 (0.90, 2.03) | **2.01 (1.29, 3.12)** | **1.92 (1.38, 2.67)** | 1.28 (0.60, 2.74) | **2.24 (1.18, 4.25)** |
| **Product use frequency** |  |  |  |  |  |  |
| Occasional | REF | REF | REF | REF | REF | REF |
| Frequent | **2.88 (1.48, 5.60)** | 1.24 (0.82, 1.88) | **3.15 (1.92, 5.17)** | 1.00 (0.63, 1.58) | **4.74 (1.65, 13.62)** | 0.90 (0.33, 2.44) |
| **Province of residence** |  |  |  |  |  |  |
| Québec | REF | REF | REF | REF | REF | REF |
| British Columbia | 0.05 (0.01, 0.32) | 0.36 (0.08, 1.56) | 0.62 (0.29, 1.35) | 0.71 (0.39, 1.29) | 0.48 (0.12, 2.05) | 0.65 (0.20, 2.13) |
| Prairie provinces | 0.11 (0.02, 0.70) | 0.82 (0.19, 3.63) | 0.71 (0.36, 1.43) | 0.82 (0.50, 1.35) | 0.55 (0.16, 1.96) | 0.77 (0.26, 2.29) |
| Ontario | 0.28 (0.05, 1.61) | 0.82 (0.19, 3.63) | 0.86 (0.47, 1.26) | 0.77 (0.47, 1.26) | 0.63 (0.19, 2.13) | 0.43 (0.15, 1.24) |
| Atlantic provinces | 0.16 (0.02, 1.13) | 0.59 (0.13, 2.74) | 0.55 (0.27, 1.12) | 0.66 (0.39, 1.11) | 0.50 (0.12, 2.05) | 0.52 (0.16, 1.67) |
| **Age** |  |  |  |  |  |  |
| MLA-25 | **11.54 (3.24, 41.17)** | **2.90 (1.18, 7.11)** | 2.35 (0.95, 5.80) | **2.10 (1.11, 3.97)** | 2.51 (0.62, 10.11) | 1.41 (0.46, 4.36) |
| 26-35 | **7.13 (2.74, 18.57)** | **2.38 (1.24, 4.54)** | **2.06 (1.00, 4.21)** | 1.08 (0.62, 1.88) | **5.24 (1.62, 16.89)** | 1.97 (0.76, 5.12) |
| 36-45 | **2.94 (1.16, 7.49)** | 1.10 (0.64, 1.89) | 1.61 (0.74, 3.51) | 1.29 (0.76, 2.21) | 3.80 (0.99, 14.45) | 2.01 (0.66, 6.14) |
| 46-55 | 1.14 (0.43, 3.05) | 0.93 (0.55, 1.56) | 1.29 (0.60, 2.79) | 1.41 (0.82, 2.40) | 1.49 (0.44, 5.08) | 0.92 (0.35, 2.44) |
| 56-65 | REF | REF | REF | REF | REF | REF |
| **Sex at birth** |  |  |  |  |  |  |
| Female | REF | REF | REF | REF | REF | REF |
| Male | 1.97 (0.95, 4.07) | 0.94 (0.59, 1.51) | 1.03 (0.65, 1.62) | 1.12 (0.81, 1.55) | **2.86 (1.31, 6.27)** | 1.85 (0.94, 3.67) |
| **Ethnicity/Race** |  |  |  |  |  |  |
| Mixed/Other | **4.21 (2.02, 8.77)** | 1.74 (0.99, 3.08) | **2.65 (1.60, 4.37)** | 1.40 (0.91, 2.14) | 1.91 (0.86, 4.24) | 0.67 (0.33, 1.36) |
| White | REF | REF | REF | REF | REF | REF |
| **Highest level of Education** |  |  |  |  |  |  |
| Less than high school | REF | REF | REF | REF | REF | REF |
| High school diploma | 0.47 (0.11, 2.06) | 1.01 (0.34, 2.97) | 1.23 (0.56, 2.73) | 1.51 (0.81, 2.83) | 0.53 (0.12, 2.41) | 0.91 (0.24, 3.44) |
| Some college or technical vocation | **0.20 (0.05, 0.82)** | 1.15 (0.42, 3.16) | 1.15 (0.55, 2.38) | 1.53 (0.85, 2.75) | 0.65 (0.16, 2.63) | 1.57 (0.45, 5.45) |
| Bachelor’s degree or higher | 0.51 (0.12, 2.18) | 1.47 (0.49, 4.39) | **2.80 (1.30, 6.04)** | **1.96 (1.03, 3.74)** | 1.12 (0.26, 4.82) | 1.98 (0.53, 7.45) |
| **Income adequacy** |  |  |  |  |  |  |
| Very difficult/Difficult | REF | REF | REF | REF | REF | REF |
| Neither easy nor difficult | 1.05 (0.46, 2.39) | 1.12 (0.69, 1.81) | 0.80 (0.47, 1.34) | 1.05 (0.71, 1.55) | 0.88 (0.35, 2.22) | 1.59 (0.76, 3.33) |
| Easy/Very easy | **2.74 (1.23, 6.11)** | 1.49 (0.85, 2.61) | 1.04 (0.59, 1.83) | 1.05 (0.69, 1.61) | 2.39 (0.92, 6.19) | 1.98 (0.86, 4.55) |
| **Survey device** |  |  |  |  |  |  |
| Smartphone | 0.95 (0.47, 1.90) | 0.91 (0.58, 1.44) | 1.06 (0.67, 1.69) | 0.99 (0.69, 1.41) | 1.14 (0.55, 2.39) | 1.21 (0.65, 2.25) |
| Tablet | 0.22 (0.02, 2.72) | 0.48 (0.20, 1.13) | 1.23 (0.46, 3.30) | 0.77 (0.33, 1.78) | 0.17 (0.01, 3.27) | 0.37 (0.11, 1.24) |
| Computer | REF | REF | REF | REF | REF | REF |

Bolded values indicate significance at p<0.05.
